# Deafness and depression in the workplace: is there an association?

**DOI:** 10.1192/j.eurpsy.2024.1123

**Published:** 2024-08-27

**Authors:** W. Ayed, D. Brahim, I. Yaich, C. Bensaid, L. Houissa, N. Mechergui, H. Bensaid, M. Mersni, G. Bahri, I. Youssef, M. Bani, N. Bram, N. Ladhari

**Affiliations:** ^1^Occupational pathology and fitness for work, Charles Nicolle Hospital; ^2^Forensic Psychiatry department, Razi Hospital, La Manouba; ^3^Occupational pathology and fitness for work department Charles Nicolle hospital, Faculty of medicine of Tunis, Tunis El Manar University, Tunis, Tunisia

## Abstract

**Introduction:**

Chronic exposure to damaging noise can lead to hearing loss . People suffering from hearing problems find it increasingly difficult to communicate and become withdrawn. This lack of contact can lead to the onset of anxiodepressive disorders .

**Objectives:**

To study the epidemiological and clinical particularities of hearing loss in patients with psychoaffective disorders.

To study the impact of this association on the medical aptitude for work.

**Methods:**

Retrospective descriptive study of depressive patients with hearing loss who consulted the Occupational Medicine Department at Charles Nicolle Hospital over a six-year period from January 2016 to November 2022.

**Results:**

Out of 150 patients with hearing loss who consulted our service, 10 patients had an axio-dépressive disorder . Seven were men and three were women. The mean age was 43 ± 5 years and the mean job seniority was 11 years [3-20]. they belonged to the telecommunications (n=6), industry (n=2), printing(n=1), and transport sectors (n=1) . The job positions were : teleconsultant (n=6), operator machine (n=3) and driver (n=1)  the symptoms presented by the patients were hearing loss (n=4), otalgia (n=1) , diziness (n=1), tinnitus(n=1) . The average time to onset of symptoms was 13±8 years [1-35] . The hearing deficits presented by the patients were: sensorineural hearing loss (n=7), mixed hearing loss (n=1) and conductive hearing loss (n=2). The mean of Hearing loss were 34±9 dB in the right ear and 34±6 dB in the left ear . A declaration of the deafness as an occupational disease was indicated in two of the cases. the univariate statistical study showed that anxiety-depressive disorders were associated with tinnitus (p=0,036,OR=4,2[0,99-17,659]) and the position of teleconsultant (p=0,009,OR=5,622[1,338-23,627] . Eviction from exposition to noise was indicated in seven cases

**Conclusions:**

According to our study, hearing loss in patients with anxio-depressice disordes is associated with tinnitus and teleconsultant job position . Early screening early screening of people at risk is recommended.

**Disclosure of Interest:**

None Declared

